# Modeling, Simulation, and Techno-Economic Assessment of a Spent Li-Ion Battery Recycling Plant

**DOI:** 10.3390/ma18153715

**Published:** 2025-08-07

**Authors:** Árpád Imre-Lucaci, Florica Imre-Lucaci, Szabolcs Fogarasi

**Affiliations:** 1 Department of Chemical Engineering, Faculty of Chemistry and Chemical Engineering, Babeş-Bolyai University, RO-400028 Cluj Napoca, Romania; arpad.imre@ubbcluj.ro; 2 Institute for Interdisciplinary Research in Bio-Nano-Science, Babeş-Bolyai University, RO-400271 Cluj Napoca, Romania

**Keywords:** spent li-ion batteries, recycling, modeling, simulation, metal recovery

## Abstract

The literature clearly indicates that both academia and industry are strongly committed to developing comprehensive processes for spent Li-ion battery (LIB) recycling. In this regard, the current study presents an original contribution by providing a quantitative assessment of a large-scale recycling plant designed for the treatment of completely spent LIBs. In addition to a concept of the basic process, this assessment also considers a case study of a thermal integration and CO_2_ capture subsystem. Process flow modeling software was used to evaluate the contribution of all process steps and equipment to overall energy consumption and to mass balance the data required for the technical assessment of the large-scale recycling plant. To underline the advantages and identify the optimal novel process concept, several key performance indicators were determined, such as recovery efficiency, specific energy/material consumption, and specific CO_2_ emissions. In addition, the economic potential of the recycling plants was evaluated for the defined case studies based on capital and O&M costs. The results indicate that, even with CO_2_ capture applied, the thermally integrated process with the combustion of hydrogen produced in the recycling plant remains the most promising large-scale configuration for spent LIB recycling.

## 1. Introduction

Based on current trajectories, it is estimated that, on a global scale, the LIB industry will reach a capacity of 1000 GWh in 2026, corresponding to almost USD 140 billion, which is almost four times the value reported in 2019 and only half of the capacity predicted for 2030 [1,2,3]. It is undeniable that LIB production capacity is on an ascendant trajectory, increasing from year to year in correlation with other industrial sectors, which boosts energy and raw material consumption to a level that clearly cannot be economically sustained by current supplies without a proper recycling process [4,5,6]. From the demand and price evolution of key energy transition minerals, it can be observed that, in just five years, the market increased by 200% to USD 320 billion last year [4,7]. Clearly, the gap between demand and supply deepens, and production costs will significantly increase due to increases in raw materials’ uncertainty and unavailability.

However, the most significant risk for LIB production is not related to the unforeseen market impact of natural resources availability dynamics, but to the continuous and unpredictable decline of ore quality, which involves more waste, higher emissions, and altogether increasing exploration costs [4,5,7,8]. For instance, Jose–Luis et al. reported a decline in copper ore grade by an average of 25% in Chile between 2003 and 2013, leading to an increase in energy costs by almost 50% [9]. Other publications indicate a decrease in copper and nickel ore quality by almost 28% between 2010 and 2017, accompanied by an increase in tailings and waste rock output by 13% and 37%, respectively [4,10]. The increasing processing cost of lower-grade ores means that raw material costs will make up a larger share of the total cost of LIB production, despite the fact that costs dropped from 1100 USD/kWh in 2010 to 150–180 USD/kWh in 2022 [8,11,12]. For instance, the share of the costs of the cathode material in batteries increased significantly from under 5% in 2015 to 20% in 2021, and, in just one year, reached 40%. This is most apparent for cobalt-rich LIBs [13], but, in general, active cathode materials account for an average of 34% of the total battery cell costs and an average of 50% [13] of the total material costs, greatly exceeding the costs associated with other battery components [14,15,16].

Different assessments have revealed that the cathode material is not only the costliest component in newly manufactured LIBs, but, at the same time, is the most important part of spent LIBs because it has the highest recycling potential from both technical and economic points of view [17]. So, regarding the sustainability of large-scale LIB production, recycling, and recovery, this material is mandatory in order to cover the necessities of the present without compromising the future [18,19]. According to Costa et al. and Dewulf et al., the cost benefit of LIB recycling goes beyond just conserving the primary reserves of critical minerals. This is because the 51% decrease in virgin materials used includes the reduction in energy vectors associated with LIB production as well, and it can prevent from 10% to 30% of the production-related environmental impact [20,21]. In addition, companies need to move towards a more circular economic production concept instead of the linear one that is most often applied, in which the obtained products are fed into the production line of fresh LIBs. Circular economic production has untapped economic potential for recycling plants that could significantly increase the profitability of companies and reduce the market risks associated with the cobalt/nickel content of SLIBs, as well as the price fluctuations of recovered metals [13,18]. For instance, it is a great option in the case of the Chinese company BRUNP, which is a subsidiary of the Chinese LIB manufacturer CATL, to couple the recycling and production facilities of the two companies, greatly increasing their economic stability. In addition to the fact that using recycled materials instead of virgin ones halves production costs, Thompson et al. found that producing metal oxides leads to 50–60% cell cost recovery in comparison to pure metallic Co, Mn, and Ni, which can generate a maximum of 5% of the cell cost [14].

Therefore, recycling spent LIBs can contribute to environmental preservation, inhibit the depletion of primary resources, diminish the amount of waste, and ultimately yield economic advantages, thus promoting sustainable development [20,22]. Still, meeting this ambitious target requires more than a series of policies, measures, and potential secondary resources; it needs massive research and innovation effort to ensure the development and large-scale deployment of ground-breaking, cost-effective, and eco-friendly spent LIB recycling technologies [23]. 

It is well-known that the mainstream recycling options include pyrometallurgy, hydrometallurgy, and direct recycling. Recent results have revealed that pyrometallurgical techniques, such as smelting, roasting, and pyrolysis, are costly and are a major source of greenhouse gas emissions [24]. Moreover, metals with lower melting points, like lithium and aluminum, are frequently lost in the slag phase, increasing the complexity and cost of recovery [17]. Conversely, hydrometallurgical recycling is a more sustainable and environmentally friendly alternative, having the potential to selectively extract metals from LIBs using processes such as leaching, solvent extraction, and chemical precipitation [25]. Compared to pyrometallurgy, hydrometallurgical methods are more energy efficient, less capital intensive, and are better at recovering lithium and other transition metals that are often lost in high-temperature processes [26]. As another option, direct recycling methods, such as cathode-to-cathode, mechanical, electrochemical, and cathode-healing technologies, offer advantages like streamlined processing, improved material quality, and lower costs [15]. However, they are typically integrated into the mechanical or hydrometallurgical steps, which can complicate the process.

A common feature among most studies in the literature is the presentation of potentially applicable process concepts, but their performance has only been evaluated in laboratory environments [27,28]. According to Dobó et al., even the existing large-scale industrial processes applied for the recycling of spent LIBs face several issues and need improvements because they were initially developed for extractive cobalt or nickel metallurgy, which are normally adjusted, but are not dedicated, to LIB recycling [17,29]. It is well known that recycling companies have difficulty meeting the stipulated 50% mass recovery required by Directive 2012/19/EU, especially in the case of Li, not to mention that the applied pyrometallurgical options are highly energy and cost intensive [24,30]. Another important problem concerns the superficial and incomplete processing of spent LIBs in many studies, focusing only on some material fractions or specific process steps, without offering a comprehensive techno-economic assessment [31,32]. 

To fill the existing gap in the literature and promote the deployment of LIB recycling technologies, the current study defines an overall process and identifies the technical performance not for individual subsystems operating at the laboratory scale, but for integrated large-scale recycling plants that include all essential subsystems. This approach leads to overall conclusions regarding variations in the technical performance of integrated industrial-scale recycling plants and provides necessary data for future environmental assessments of this process.

## 2. Materials and Methods

### 2.1. Description of the Mathematical Models Developed for the Recovery Process of Metals from Spent LIBs

**Figure 1:**
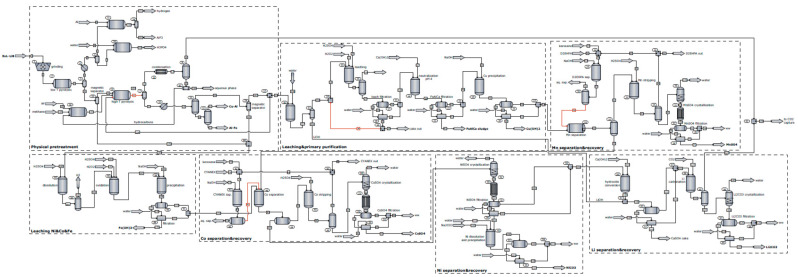
Process flow diagram of the spent Li-ion battery recycling plant.

Figure 1 presents the mathematical model developed for the recycling process of spent LIBs with processing capacities of 1314 kg/h, which fall within the range reported for other industrial facilities in the literature. As shown, the mathematical model involves sequential processing of spent LIBs (lithium–nickel–manganese–cobalt oxide cathode material), leading to the recovery of nickel and lithium in the form of carbonates and manganese and cobalt in the form of sulfates. In addition, the recycling plant generates by-products that are either used for process integration or which are valorized alongside the main products. The process is divided into the following subsystems:

1. **The mechano-thermal treatment of spent LIBs:** This is the first stage of the process, and its main purpose is the disassembly and sorting of spent LIBs into different material fractions that are processed through thermal treatments to dissolve the components of interest. The material obtained in the grinding and sieving step undergoes thermal treatment in two stages: (i) at 370 °C, the separation of the electrolyte and its treatment occurs; (ii) at 797 °C, the plastic fractions are converted by pyrolysis to combustible products, with a parallel partial carbothermic reduction in oxide materials. This stage also involves the separation of magnetic metals (Ni, Fe, Co) from non-magnetic materials, which are then transferred to the corresponding subsystems for further processing.
2. **Dissolution and purification:** This subsystem is designed for the processing of the non-magnetic material stream obtained from the mechano–thermal treatment of spent LIBs. The dissolution of oxides takes place in an acidic environment with a reducing agent (oxalic, formic, citric acid, H_2_O_2_) depending on the case study, followed by the separation, in an alkaline environment, of the solution rich in sulfates of Mn, Ni, and Co from other secondary products. Before oxide dissolution, lithium is selectively extracted as a LiOH solution by washing it solid stream with water.
3. **Separation and recovery of manganese:** In this subsystem, the selective extraction of MnSO_4_xH_2_O takes place in an alkaline environment using kerosene and Di-(2-Ethyl Hexyl) phosphoric acid as solvents. To reduce solvent consumption, the subsystem involves their regeneration in a sulfuric acid medium and subsequent recirculation in the process.
4. **Dissolution of magnetic metals:** The stream of magnetic metals (Ni, Fe, Co) is treated with sulfuric acid in adiabatic conditions to obtain the corresponding sulfate solutions and to produce H_2_ usable for thermal energy generation in the process.
5. **Separation and recovery of cobalt:** The extraction process is similar to that used for MnSO_4_xH_2_O, with the difference that Di-(2-Ethyl Hexyl) phosphoric acid is replaced by CYANEX, which is selective for Co^2+^. In addition, obtaining solid CoSO_4_ also involves a crystallization–recrystallization and filtration step.
6. **Separation and recovery of nickel:** From the aqueous solutions of sulfates resulting in the last two stages, a solution of NiSO_4_ is obtained by crystallization–recrystallization and filtration, which is treated with a solution of Na_2_CO_3_ to precipitate and separate solid NiCO_3_.
7. **Separation and recovery of lithium:** In the last subsystem of the process, Li_2_CO_3_ is obtained through the following two consecutive steps: (i) obtaining the LiOH solution by treating the exhausted solution from the nickel recovery subsystem with Ca(OH)_2_ and mixing the filtrate with the LiOH solution from the dissolution and purification subsystem; and (ii) carbonation of the LiOH solution, followed by crystallization and filtration of Li_2_CO_3_.

For the evaluation of the technical performance of the spent LIB recycling plant, the following case studies were considered:

- **Case 1**: Thermally not-integrated process.
- **Case 2:** Thermally integrated process with H_2_ combustion.

Both case studies were assessed for the impact of CO_2_ capture as well.

### 2.2. Description of the Mathematical Model Developed for the CO_2_ Capture Process

**Figure 2:**
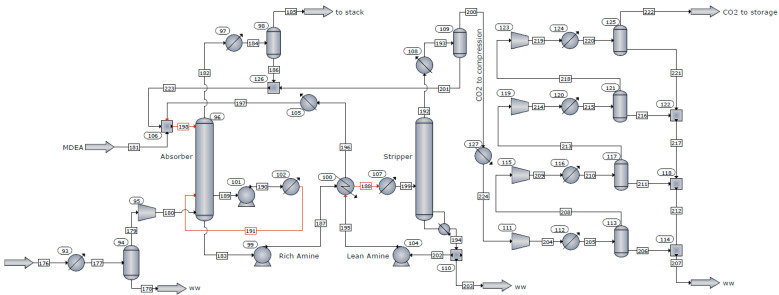
Process flow diagram of the CO_2_ captures process.

Considering the importance of CO_2_ capture in the industrial sector, the recycling plant of spent LIBs was coupled with a post-combustion CO_2_ capture **system based on an amine absorption process**. According to Figure 2, the CO_2_ capture process involves the following three major steps: (i) Absorption of CO_2_ into the lean amine solution at a temperature of approximately 35–55 °C and a pressure of 1.05 bar. (ii) The rich amine-CO_2_ stream is pumped and heated to a temperature of about 100–120 °C using the hot mass of solvent from the bottom of the desorption column. After preheating, the CO_2_-rich stream is passed to the desorption column where the solvent is regenerated using thermal energy provided by the recycling plant. (iii) The CO_2_ stream is dried and compressed in four stages up to the storage pressure of 122 bar.

### 2.3. Methodology and Basic Assumptions for Technical Assessment

The technical performance of the metal recovery processes from spent LIBs was carried out by simulating and optimizing the processes using the process flow modeling software SuperPro Designer (version: v14), specific to chemical engineering. In the simulations, chemical and phase equilibrium conditions were defined based on the Gibbs free energy minimization model. The calculations reached a steady state solution and were stopped (converged) when the solution no longer changed significantly with further iterations, or, in other words, when the changes in key variables between iterations were below 10^−6^. Assumptions included a pressure loss of 1% in heat exchangers, a minimum temperature difference of 10 °C for thermal integration, and a pressure drop of 46 mbar in the barometric condenser. To identify the optimal systems for metal recovery from spent LIBs, the technical performance of the thermally integrated/non-integrated technological variants, with and without CO_2_ capture, were compared under steady-state conditions. In addition, the impact of the type of reducing agent (H_2_O_2_, C_6_H_8_O_7_, HCOOH, and H_2_C_2_O_4_) on the performance of the spent LIB recycling plant was assessed.

### 2.4. Methodology and Basic Assumptions for Economic Assessment

For the economic evaluation of the developed processes, SuperPro Designer was also used. This software enables the development and application of economic models based on the mathematical representation of the recycling facility, while taking into account specified economic parameters and context. In addition, SuperPro Designer provides a comprehensive database of material costs, utility costs, equipment cost models based on industrial experience, and cost models for treating spent streams/waste. All of these aspects ensure that the results are grounded in industrial practices and are consistent with data from the specialized literature.

The methodology for estimating capital costs involved calculating the cost of each equipment using correlation equations presented in the literature, whose general form is shown in Equation (1):

(1) $$C=C_{0}*{\left(\frac{Q}{Q_{0}}\right)}^{M}$$

where C_0_ and Q_0_ represent the reference costs and sizes for each equipment and M is a parameter dependent on the equipment type. C and Q represent the actual capital costs and the sizes of the equipment in the recycling facility. The SuperPro Designer database contains all the parameters required for cost calculations, ensuring that the economic evaluation is up to date for the year 2025.

The capital costs (equipment), along with other economic indicators listed in Table 1, were used to calculate the total investment costs.

In addition to capital costs, operating and maintenance costs (O&M) were integrated into the economic model of the developed technologies and were calculated by summing the following components:

- Material costs (MCs): Calculated based on the price and quantity of the materials involved.
- Labor costs (LCs): An average rate of 30.75 USD/h was applied.
- Facility-dependent costs (FDCs): Including maintenance costs (6% of DFC), insurance, taxes, and other production-related expenses (5% of DFC).
- Quality control and assurance costs (QC/QA), which are 2.5% of LCs.
- Waste and spent stream processing costs (WT) depend on the type and quantity of the materials and the cost of the treatment process.
- Utility costs: Electricity and thermal agents.

## 3. Results and Discussions

### 3.1. Technical Performance of the Spent LIB Recycling Process

Based on material balance data, the recovery yield and production rate of the main products in the spent LIB recycling process were determined. Since the values of these performance indicators are very similar, being almost identical for the technological variants using different reducing agents, only the average values are presented in Table 2. The results indicate a high performance because, apart from the recovery yield of cobalt, they exceed 80% and, in some situations, even 90%.

Clearly, achieving these performance levels requires the supply of raw materials in different amounts and ratios to the process. According to the data presented in Table 3, the specific consumption of raw materials in kg/kg LIBs is the highest for the thermally not-integrated process (Case 1) in which the combustion of additional amounts of CH_4_, to generate the required thermal energy, leads to increased air consumption. Almost 80% of the total specific consumption of raw materials is attributed to the air used in the combustion process of CH_4_. In comparison, the thermally integrated process (Case 2), which uses less CH_4_ and implicitly air, is characterized by a specific air consumption of nearly five times lower than for Case 1. This reduction also results in a 250% decrease in the total specific raw material consumption. 

Regarding the specific consumption of raw materials in kg/kg product, across different subsystems of the spent LIB recycling process, the results in Table 4 indicate the highest values for the subsystem used for the dissolution of magnetic metals (Subsystem 4), followed by the subsystems defined for the separation and recovery of Ni (Subsystem 6), and finally for the mechano–thermal treatment of spent LIBs (Subsystem 1). Since the values are relatively similar across for the case studies involving different reducing agents, Table 4 only presents the data for the one with oxalic acid. In contrast, the subsystems for the dissolution and purification of oxide materials (Subsystem 2), as well as the one for the recovery of Li_2_CO_3_ (Subsystem 7), exhibit the lowest specific raw material consumption.

On the other hand, the performance profile differs significantly when considering the specific thermal energy consumption determined based on the energy balance data. The results in Table 5 indicate that, among all of the subsystems of the spent LIB recycling plant, only the dissolution and purification subsystem is an energy generator, even for the thermally not-integrated process. In contrast to the specific raw material consumption, the subsystem responsible for the separation and recovery of cobalt exhibits the poorest performance, as it consumes more than 50% of the total thermal energy supplied in Case 1. The next most energy-intensive subsystems are those used for the separation and recovery of nickel and manganese. It is important to mention that the thermal integration of the process massively reduces the consumption of both thermal energy and CH_4_, resulting in values that are ten times lower. Even if the specific consumption of CH_4_ does not decrease to zero for the Case 2 process, it is worth noting that the system can provide 3.8 MJ of thermal energy with each kg of recycled LIB, and at a relatively high temperature (380 °C).

The thermally not-integrated process stands out as the least efficient in terms of both total and specific CO_2_ emissions, as well as in the energy consumption required for CO_2_ capture for the recycling of spent LIBs. As can be seen from Table 6, this case study results in approximately 1.5 times more CO_2_ emission than in Case 2. Regarding the energy consumption for CO_2_ capture, apart from the thermally not-integrated process, it can be covered by the thermal energy generated in the recycling process of spent LIBs. Moreover, the process remains a net a thermal energy generator, even with CO_2_ capture producing 1.5 GJ/h for Case 2 which, is the most efficient in terms of recycling spent LIB, regardless of the reducer used.

### 3.2. Economic Performance of the Spent LIB Recycling Process

Economic performance was evaluated in relation to the following technological options:

- Case 1: Thermally not-integrated recycling plant without CO_2_ capture;
- Case 2: Thermally integrated recycling plant without CO_2_ capture;
- Case 3: Thermally not-integrated recycling plant with CO_2_ capture;
- Case 4: Thermally integrated recycling plant with CO_2_ capture.

It is important to note that the economic analysis for all four case studies is based solely on the use of oxalic acid as the reducing agent in the dissolution and purification stage because the technical and economic differences between the reducing agents were found to be negligible.

Based on the material/energy balance data and the capacity of the equipment involved, the total investment costs for the four case studies of the spent LIB recycling process were determined. Table 7 presents the total investment costs for the recycling of spent LIBs without the capture of CO_2_. As the results reveal, the largest investment is associated with the subsystem responsible for the mechanical–thermal processing of used LIBs, primarily due to the energy-intensive processes and the extreme operating conditions required by the equipment in this subsystem. It can be observed that approximately 90% of the total investment consists of direct costs, while only 10% corresponds to equipment costs.

As expected, according to the results in Table 8, the introduction of the CO_2_ capture stage increases both the total and specific investment costs by approximately 5%, regardless of whether the process is a thermally integrated or thermally not-integrated process. However, no changes occur in the investment costs of the other subsystems, as the integration of the CO_2_ capture subsystem does not affect the number, type, or capacity of the equipment in other subsystems. Thus, a minimum investment of USD 2283 for the processing of 1000 kg of LIB is associated with the thermally integrated technological variant without CO_2_ capture (Case 1), and a maximum investment of USD 2390 occurs in the case of the thermally not-integrated process with CO_2_ capture (case 3).

In parallel with the total investment costs, the O&M costs for the recycling of spent LIBs were also evaluated for the four case studies. The results in Table 9 show that the highest costs are associated with material consumption and the labor force required for the operation of the facility and the management of the processes. Regarding the material costs, the highest share is H_2_SO_4_ (~48% of MC), followed by NaOH (~10% of MC) and Ca(OH)_2_ (~12% of MC), considering that, regardless of the case study, these materials are essential for the separation and regeneration of solvents used for the recovery of manganese, cobalt, and lithium, as well as for the neutralization of some waste streams. 

Compared to total investment costs, O&M costs exhibit a more pronounced differentiation between the processes with and without thermal integration. The costs related to Case 2 are approximately 7% lower than those for Case 1, primarily due to the reduction in thermal agents. For this reason, the utility costs decrease on average by 70%, with the most obvious variations being in the recovery stages of nickel, cobalt, and lithium, where crystallization is optimized by thermal integration.

The introduction of a CO_2_ capture stage also negatively impacts O&M costs, which increase by 20% compared to non-capture scenarios. These additional costs are generated by the high thermal agent consumption used for the regeneration of the solvent used in the CO_2_ capture subsystem. Therefore, the cost of utilities increases three times for the thermally not-integrated process and six times for the thermally integrated one. However, the cost of utilities remains lower for the thermally integrated process, being only 40% of the value for the thermally not-integrated process. It can also be seen from the results presented in Table 10 that the CO_2_ capture subsystem accounts for 90% to the total utility costs.

In addition, the production costs and profitability of the spent LIB recycling process were evaluated over a 15-year plant lifetime, assuming a 3-year construction period and excluding any subsidies or acquisition costs for spent LIBs. According to Table 11, all technological options are profitable and promising, considering the values of the economic indicators. As expected, the highest specific production cost is attributed to cases with CO_2_ capture and without thermal integration, while the highest specific net profit is achieved in processes without CO_2_ capture. According to the results, in all cases, the investment is recovered in less than two years after the recycling plant reaches full-capacity production.

## 4. Conclusions

The obtained results prove that the developed, modeled, and simulated recycling plant is adequate and efficient for the sequential processing of spent LIBs, enabling the recovery of nickel and lithium in the form of carbonates and manganese and cobalt in the form of sulfates. Additional by-products such as CaSO_4_ and Fe_2_(SO_4_)_3_ are also generated, which may be valorized in other industrial sectors. It was found that, in the best operating conditions, the recovery rate of critical materials exceeds 80% and the purity of the obtained main products was more than 99%, making them suitable for new LIB production or other industrial applications. Based on the energy balance data, it can be concluded that, beyond its technical potential, the process remains a net thermal energy generator, even with CO_2_ capture, producing 1.5 GJ/h for thermally integrated configurations. As an overall conclusion, it can be stated that the technological configuration with H_2_ combustion is the most efficient in terms of recycling spent LIBs, regardless of the inclusion of CO_2_ capture. Given the current findings, future studies may rethink and redesign some key steps and investigate other case studies, as well improve the technical performance of the spent LIB recycling plant. Moreover, it is necessary to apply material flow analyses and life cycle assessments for the developed conceptual recycling plant to facilitate global benchmarking against alternative recycling methods.

## Figures and Tables

**Table 1:** Methodology for estimating total investment costs.

No.	Economic Indicator	Definition
1	**Direct costs (DC)**	
1.1	Capital costs (PC)	Σ C (according to Equation (1))
1.2	Installation costs (CI)	38% of PC
1.3	Piping **and** connections costs	35% of PC
1.4	Process instrumentation costs	40% of PC
1.5	Insulation costs	3% of PC
1.6	Electrical installation costs	10% of PC
1.7	Building costs (interior fittings)	45% of PC
1.8	Production platform development costs	15% of PC
1.9	Auxiliary installation costs	40% of PC
2	**Indirect costs (IC)**	
2.1	Engineering purchases (EC)	25% of DC
2.2	Construction costs (CC)	35% of DC
3	**Other costs (OC)**	
3.1	Contract fees (CF)	5% of (DC + IC)
3.2	Contingency costs (Con)	10% of (DC + IC)
4	**Fixed capital (DFC)**	
5	**Working capital for 30 days (CL)**	
6	**Startup costs (CP)**	5% of DFC
7	**Total investment costs (TIC)**	TIC = DFC + CL + CP

**Table 2:** Recovery yields and production rates for the main products of the spent LIB recycling plant.

Product	MnSO_4_xH_2_O	CoSO_4_x7H_2_O	NiCO_3_	Li_2_CO_3_	Fe(OH)_3_	Graphite	Al	Cu
**Production rate, kg/h**	206.63	299.03	208.76	131.81	98.77	189.71	296.25	101.5
**Recovery yield, %**	84.09	79.26	82.61	94.84	98.21	87.50	93.24	90.91

**Table 3:** Specific consumption of raw materials in kg/kg LIB for the spent LIB recycling process.

Raw Material	H_2_O	CH_4_	H_2_SO_4_	Na_2_CO_3_	NaOH	air	Ca(OH)_2_	CO_2_	TOTAL
**Consumption, kg/h**	2544	120	1019.7	211.98	424.76	3500	137	98.5	-
**Case 1**	1.94	0.09	0.78	0.16	0.32	11.39	0.10	0.07	14.85
**Case 2**	1.94	0.09	0.78	0.16	0.32	2.66	0.10	0.07	6.17

**Table 4:** Specific consumption of raw materials in kg/kg product for different subsystems of the spent LIB recycling process using oxalic acid.

No. Subsystem	1	2	3	4	5	6	7
**TOTAL, kg/h**	4959.5	801.86	727.10	1145.69	693.60	861.98	272.50
**W, kg/kg**	3.77	2.08	3.52	4.61	2.32	4.13	2.07

**Table 5:** Thermal energy balance for the recycling process of spent LIB using oxalic acid.

No. Subsystem	Parameters of Energy Flows	Thermal Energy Generated			Thermal Energy Consumed			TOTAL Consumption, MJ/h	Specific Consumption, MJ/kg	Equivalent Consumption, kg CH_4_/h
		1	2	3	1	2	3			
**1**	**T, °C**	120	40	380	370	797				
	**Q, MJ/h**	−488	−51	−11,269	1173	76		1249	0.95	25
**2**	**T, °C**	60								
	**Q, MJ/h**	−1704						0	0	0
**3**	**T, °C**	24			24	40	25			
	**Q, MJ/h**	−1687			1469	271	419	2159	10.45	43
**4**	**T, °C**	32			40					
	**Q, MJ/h**	−1713			700			700	2.82	14
**5**	**T, °C**	24			24	40	25			
	**Q, MJ/h**	−1395			1384	5057	582	7023	23.49	140
**6**	**T, °C**	40	110		110					
	**Q, MJ/h**	−258	−583		2129			2129	10.20	43
**7**	**T, °C**	35			93					
	**Q, MJ/h**	−112			387			387	2.94	8
**Case 1**								**13,646**		273
**Case 2**	**T, °C**	380			370	797				
	**Q, MJ/h**	−781			1173	76		**1249**	0.95	25

**Table 6:** Total and specific CO_2_ emissions, respectively; the average energy consumption of the CO_2_ capture process for the recycling of spent LIBs.

Process Type	Case 1		Case 2	
Reducing agent	Total, kgCO_2/_h	kg CO_2_/kg LIB	Total, kgCO_2/_h	kg CO_2_/kg LIB
**H_2_O_2_**	1402	1.07	652	0.50
**C_6_H_8_O_7_**	1418	1.08	667	0.51
**HCOOH**	1425	1.08	675	0.51
**H_2_C_2_O_4_**	1449	1.10	698	0.53
**CO_2_ capture energy consumption_,_ GJ/h**	4.73		2.24	

**Table 7:** Total investment costs for recycling used LIBs without CO2 capture.

	Subsystem	Capital Cost (CC )	Direct Cost (DC)	Working Capital (WC)	Start-Up Cost (SC)	Total Investment Cost	Specific Investment Cost
		kUSD					USD/t LIBs
**Case 1**	Mechanical-thermal treatment of spent LIBs	1393	8330	347	416	9094	539
	Dissolution and purification	848	5100	373	255	5728	339
	Manganese separation and recovery	983	5877	391	294	6562	389
	Cobalt separation and recovery	716	4260	338	213	4810	285
	Nickel separation and recovery	554	3332	204	167	3702	219
	Lithium separation and recovery	603	3612	173	181	3966	235
	Magnetic metal dissolution	676	4067	392	203	4662	276
	Total, kUSD	5771	34,577	2219	1729	**38,525**	
	Total, USD/t LIBs	342	2049	131	102		**2283**
**Case 2**	Mechanical-thermal treatment of spent LIBs	1417	8482	330	424	9236	547
	Dissolution and purification	848	5100	373	255	5728	339
	Manganese separation and recovery	983	5877	385	294	6555	389
	Cobalt separation and recovery	716	4260	331	213	4804	285
	Nickel separation and recovery	554	3332	159	167	3658	217
	Lithium separation and recovery	603	3612	151	181	3943	234
	Magnetic metal dissolution	676	4067	392	203	4662	276
	Total, kUSD	5796	34,729	2121	1736	**38,586**	
	Total, USD/t LIBs	343	2058	126	103		**2287**

**Table 8:** Total investment costs for recycling used LIBs with CO_2_ capture.

	Subsystem	Capital Cost (CC)	Direct Cost (DC)	Working Capital (WC)	Start-Up Cost (SC)	Total Investment Cost	Specific Investment Cost
		kUSD					USD/t LIB
**Case 3**	Mechanical-thermal treatment of spent LIBs	1393	8330	309	416	9056	537
	Dissolution and purification	848	5100	373	255	5728	339
	Manganese separation and recovery	983	5877	391	294	6562	389
	Cobalt separation and recovery	716	4260	338	213	4810	285
	Nickel separation and recovery	554	3332	204	167	3702	219
	Lithium separation and recovery	603	3612	173	181	3966	235
	Magnetic metal dissolution	676	4067	392	203	4662	276
	CO_2_ capture	209	1302	474	65	1842	109
	Total, kUSD	5981	35,879	2655	1794	**40,328**	
	Total, USD/t LIBs	354	2126	157	106		**2390**
**Case 4**	Mechanical-thermal treatment of spent LIBs	1417	8482	292	424	9198	545
	Dissolution and purification	848	5100	373	255	5728	339
	Manganese separation and recovery	983	5877	385	294	6555	389
	Cobalt separation and recovery	716	4260	331	213	4804	285
	Nickel separation and recovery	554	3332	159	167	3658	217
	Lithium separation and recovery	603	3612	151	181	3943	234
	Magnetic metal dissolution	676	4067	392	203	4662	276
	CO_2_ capture	178	1108	358	55	1521	90
	Total, kUSD	5974	35,837	2441	1792	**40,069**	
	Total, USD/t LIBs	354	2124	145	106		**2375**

**Table 9:** O&M costs for recycling spent LIBs without CO_2_ capture.

	Subsystem	Materials	FDC	Labor Force	QC/QA	Utilities	WT	Total Cost of O&M	Specific Cost of O&M
		kUSD/year							USD/t LIBs
**Case 1**	Mechanical-thermal treatment of spent LIBs	259	111	1218	30	401	1943	3962	235
	Dissolution and purification	2480	960	1327	199	44	249	5259	312
	Manganese separation and recovery	2380	100	1600	160	100	224	4564	270
	Cobalt separation and recovery	2430	805	950	142	73	263	4663	276
	Nickel separation and recovery	162	631	1041	156	505	533	3028	179
	Lithium separation and recovery	77	683	1186	178	253	392	2769	164
	Magnetic metal dissolution	3229	766	1067	160	16	<1	5238	310
	Total, kUSD/year	11,017	4056	8389	1025	1392	3604	**29,483**	
	Total, USD/t LIBs	653	240	497	61	82	214		**1747**
**Case 2**	Mechanical-thermal treatment of spent LIBs	259	113	1218	30	214	1943	3777	224
	Dissolution and purification	2480	960	1327	199	44	249	5259	312
	Manganese separation and recovery	2380	100	1600	160	29	224	4493	266
	Cobalt separation and recovery	2430	805	950	142	2	263	4592	272
	Nickel separation and recovery	162	631	1041	156	13	533	2536	150
	Lithium separation and recovery	77	683	1186	178	3	392	2519	149
	Magnetic metal dissolution	3229	766	1067	160	16	<1	5238	310
	Total, kUSD/year	11,017	4058	8389	1025	321	3604	**28,414**	
	Total, USD/t LIBs	653	241	497	61	19	214		**1684**

**Table 10:** O&M costs for recycling used LIBs with CO_2_ capture.

	Subsystem	Materials	FDC	Labor Force	QC/QA	Utilities	WT	Total Cost of O&M	Specific Cost of O&M
		kUSD/year							USD/t LIBs
**Case 3**	Mechanical-thermal treatment of spent LIBs	259	111	1218	30	401	1526	3545	210
	Dissolution and purification	2480	960	1327	199	44	249	5259	312
	Manganese separation and recovery	2380	0	1600	160	100	224	4464	265
	Cobalt separation and recovery	2430	805	950	142	73	263	4663	276
	Nickel separation and recovery	162	631	1041	156	505	533	3028	179
	Lithium separation and recovery	77	683	1186	178	253	392	2769	164
	Magnetic metal dissolution	3229	766	1067	160	16	<1	5238	310
	CO_2_ capture	1266	242	568	85	3384	176	5721	339
	Total, kUSD/year	12,283	4198	8957	1110	4776	3363	**34,687**	
	Total, USD/t LIBs	728	249	531	66	283	199		**2056**
**Case 4**	Mechanical-thermal treatment of spent LIBs	259	113	1218	30	212	1526	3358	199
	Dissolution and purification	2480	960	1327	199	44	249	5259	312
	Manganese separation and recovery	2380	0	1600	160	29	224	4393	260
	Cobalt separation and recovery	2430	805	950	142	2	263	4592	272
	Nickel separation and recovery	162	631	1041	156	13	533	2536	150
	Lithium separation and recovery	77	683	1186	178	3	392	2519	149
	Magnetic metal dissolution	3229	766	1067	160	16	<1	5238	310
	CO_2_ capture	1266	206	568	85	2099	153	4377	259
	Total, kUSD/year	12,283	4164	8957	1110	2418	3340	**32,272**	
	Total, USD/t LIBs	728	247	531	66	143	198		**1913**

**Table 11:** Production costs and profitability of the recycling process of spent LIBs.

Economic Parameter	U.M.	Case 1	Case 2	Case 3	Case 4
Total production cost	kUSD/year	29,484	28,414	34,687	32,272
Main income		23,737	23,737	23,737	23,737
Secondary income		43,732	42,616	43,831	42,616
Total income		67,469	66,353	67,568	66,353
**Specific production cost**	**USD/t LIB**	**1742**	**1678**	**2045**	**1904**
**Total specific income**		**3999**	**3933**	**4005**	**3933**
Gross profit	kUSD/year	38,085	38,039	33,055	34,234
Taxes		9521	9510	8264	8559
**Net profit**		**28,564**	**28,529**	**24,792**	**25,676**
**Specific net profit**	**USD/t LIB**	**1693**	**1691**	**1469**	**1522**
Gross margin	%	56.45	57.33	48.92	51.59
Return on investment	%	74.14	73.94	61.47	64.08
Payback period of investment	year	1.35	1.35	1.63	1.56
Internal rate of return (after paying taxes)	%	50.17	50.05	42.73	44.19

## Data Availability

The original contributions presented in this study are included in the article material. Further inquiries can be directed to the corresponding author.
